# Improving the yield and nitrogen use efficiency of hybrid rice through rational use of controlled-release nitrogen fertilizer and urea topdressing

**DOI:** 10.3389/fpls.2023.1240238

**Published:** 2023-08-24

**Authors:** Yuanyuan Sun, Xiaojuan Yuan, Kairui Chen, Haiyue Wang, Yongheng Luo, Changchun Guo, Zhonglin Wang, Chuanhai Shu, Yonggang Yang, Yanfang Weng, Xiaobo Zhou, Zhiyuan Yang, Zongkui Chen, Jun Ma, Yongjian Sun

**Affiliations:** ^1^ State Key Laboratory of Crop Gene Exploration and Utilization in Southwest China, Sichuan Agricultural University, Chengdu, Sichuan, China; ^2^ Institute of Plateau Meteorology, China Meteorological Administration, Chengdu, Sichuan, China; ^3^ Crop Ecophysiology and Cultivation Key Laboratory of Sichuan Province, Sichuan Agricultural University, Chengdu, China; ^4^ Sichuan Agricultural Machinery Research Academy, Sichuan Academy of Agricultural Sciences, Chengdu, Sichuan, China

**Keywords:** hybrid *indica* rice, controlled-release N fertilizer, urea N topdressing, grain yield, N use efficiency

## Abstract

**Introduction:**

Controlled-release fertilizers effectively improve crop yield and nitrogen use efficiency (NUE). However, their use increases the cost of crop production. Optimal management modes involving urea replacement with controlled-release N fertilizers to increase rice yield through enhanced NUE are not widely explored.

**Methods:**

Field experiments were conducted from 2017 to 2018 to determine the effects of different controlled-release N fertilizers combined with urea [urea-N (180 kg ha^-1^, N_1_)]. We used controlled-release N (150 kg ha^-1^, N_2_) as the base, and four controlled-release N and urea-N ratio treatments [(80%:0% (N_3_), 60%:20% (N_4_), 40%:40% (N_5_), or 20%:60% (N_6_) as the base with 20% urea-N as topdressing at the panicle initiation stage under 150 kg ha^-1^] to study their impact on the grain yield and NUE of machine-transplanted rice.

**Results and discussion:**

Grain yield and NUE were positively correlated with increases in photosynthetic production, flag leaf net photosynthetic rate (*P_n_*), root activity, N transport, and grain-filling characteristics. The photosynthetic potential and population growth rate from the jointing to the full-heading stage, highly effective leaf area index (LAI) rate and *P_n_* at the full-heading stage, root activity at 15 d after the full-heading stage, and N transport in the leaves from the full-heading to mature stage were significantly increased by the N_4_ treatment, thereby increasing both grain yield and NUE. Furthermore, compared with the other N treatments, the N_4_ treatment promoted the mean filling rate of inferior grains, which is closely related to increased filled grains per spikelet and filled grains rate. These effects ultimately improved the grain yield (5.03-25.75%), N agronomic efficiency (NAE, 3.96-17.58%), and N partial factor productivity (NPP, 3.98-27.13%) under the N_4_ treatment. Thus, the N_4_ treatment with controlled-release N (60%) and urea-N (20%) as a base and urea-N (20%) as topdressing at the panicle-initiation stage proved effective in improving the grain yield and NUE of machine-transplanted hybrid *indica* rice. These findings offer a theoretical and practical basis for enhancing rice grain yield, NUE, and saving the cost of fertilizer.

## 1 Introduction

Rice (*Oryza sativa* L.) is one of the major food crops worldwide (Mohanty, 2013; Sun et al., 2023a). With population growth and economic development, it is estimated that by 2030, the world’s total rice production will have to increase by 60% and 20% in China to meet demand (Peng et al., 2009; Guo et al., 2023). Applying fertilizers, especially N fertilizers, has become a key factor in increasing rice yields (Peng et al., 2006). To obtain high rice yields, the application rate of fertilizer has been greatly increased; however, the nitrogen use efficiency (NUE) has decreased (Peng et al., 2009). The average NUE worldwide is approximately 46%, whereas that in China is only 30-35% (Peng et al., 2006; Sun et al., 2023a). For this reason, many scholars have explored methods for improving the NUE through variety selection (Ma and Yuan, 2015; Li et al., 2023), straw return (Yu et al., 2022), N fertilizer application (Zhu et al., 2021), soil testing formula fertilization (Chen et al., 2021), and leaf color diagnosis (Peng et al., 1996; Pampolino et al., 2007). Further research may lead to increases in the nutrient release and regulation of controlled-release N fertilizer to meet the demand for N fertilizer during the entire growth period of crops, conserve fertilizer and labor, reduce environmental pollution, and improve NUE (Sun et al., 2020a; Lyu et al., 2021b; Chen et al., 2022). Such strategies also represent an effective method of increasing grain yield and reducing N fertilizer use (Lyu et al., 2021a; Chen et al., 2022).

Studies have also shown that the photosynthetic production of hybrid rice is high during the reproductive growth period, and the required amount of fertilizer is also high (Yang et al., 2014; Xu et al., 2021). The fertilization method that combines controlled-release N fertilizer with a “one-time basal fertilizer application” cannot meet the needs of rice growth after the heading stage (Tian et al., 2010; Ma and Yuan, 2015; Lyu et al., 2021a). Moreover, the production of controlled-release fertilizers is complex and expensive. The release rate of certain controlled-release fertilizers is slow in the early stages, which increases the difficulty of meeting the nutrient demand of rice in the early and middle stages of jointing and booting (Wu et al., 2021; Yu et al., 2022). Current research on controlled-release N fertilizers has mainly focused on the manual rice transplantation method (Peng et al., 2014; Yu et al., 2022). With continued research, relevant reports have been published on machine-transplanted (Ke et al., 2018; Lyu et al., 2021a) and direct-seeded rice (Cheng et al., 2022; Sun et al., 2022). However, under each planting mode, previous studies mainly focused on comparing controlled-release fertilizer types (Peng et al., 2014; Wu et al., 2021), application amounts (Zhu et al., 2021; AL Aasmi et al., 2022), side-deep fertilization methods (Zhu et al., 2019; Hou et al., 2021), and combined controlled-release N fertilizer and available N fertilizer under a “one-time basal fertilizer application.” (Lyu et al., 2021b; Xu et al., 2021). The demand for N fertilizer is difficult to meet throughout the growth and developmental stages of rice (Tian et al., 2010; Lyu et al., 2021a). Under the condition of mechanical transplanting, whether controlled-release N fertilizer can further improve the yield and NUE of mechanically transplanted rice, whether conventional N fertilizer can replace controlled-release N fertilizer in an appropriate proportion to reduce production costs, and whether the release of controlled-release N fertilizer nutrients and conventional N fertilizer meets the growth and development characteristics of mechanically transplanted rice relative to that of the current application level and mode of conventional N fertilizer production remain to be systematically studied.

Grain-filling, the final process of rice yield formation, determines the grain yield of rice (Huang et al., 2011; Ma and Yuan, 2015; Zhong et al., 2022). The grain yield of rice depends on the photosynthetic material production capacity (source), panicle composition traits (sink), and translocation and distribution (flow) of photosynthetic assimilates after anthesis (Lyu et al., 2021a; Li et al., 2023). Hybrid rice, especially heavy-panicle hybrid rice and super rice has a high sink capacity (Huang et al., 2011; Sun et al., 2018; Sun et al., 2022). Although the “sink” problem associated with the population and panicle morphology (more tillers, more grains per panicle, high 1000-grain weight, single panicle weight, etc.) has been resolved with these varieties (Yang et al., 2006a; Yang et al., 2014; Sun et al., 2023b), further research is required to determine whether the “source” and “flow” aspects are reasonable under controlled-release N fertilizer application to meet the N fertilizer requirements for the rice reproductive growth period, promote full grain-filling and high grain-filling, and fully exploit the potential advantages of hybrid rice varieties.

Therefore, the controlled-release fertilizer is a new type of fertilizer and has been proven effective in improving crop yield. However, the controlled-release fertilizer cannot meet the rapid nutrient demand in the early and late crop growth stages. In addition, producing this new type of fertilizer is complex and expensive. An optimal management mode wherein urea partially replaces controlled-release N fertilizers to increase rice yield through enhanced NUE has not been widely explored. Based on our previous studies (Sun et al., 2012; Yang et al., 2014; Sun et al., 2022), the present study designed different ratios of controlled-release fertilizer combined with urea-N and clarified the influences on grain yield, grain-filling characteristics, N translocation, and NUE of machine-transplanted hybrid rice, and determined the optimal ratio of controlled-release N fertilizer and urea. These findings provide a theoretical and practical basis for enhancing rice grain yield, NUE, and reducing the expenses on fertilizers.

## 2 Materials and methods

### 2.1 Study site and materials

**Figure 1:**
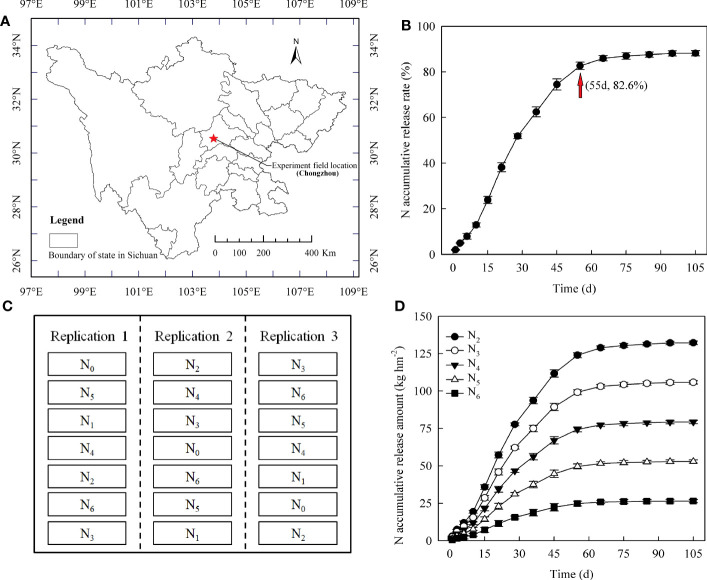
The experiment field location **(A)**, N cumulative release rate curve of controlled-release N fertilizer **(B)** immersed in water at pH 7 and 25°C, the randomized complete block design **(C)**, and N cumulative release amount curves of different controlled-release N fertilizer treatments **(D)** immersed in water at pH 7 and 25°C. N_0_: no N application; N_1_: farmer conventional urea N (180 kg ha^-1^) practice; N_2_: controlled-release N (150 kg ha^-1^) as a base; N_3_: controlled-release N (120 kg ha^-1^) as a base + urea N (30 kg ha^-1^) topdressing at panicle initiation stage; N_4_: controlled-release N (90 kg ha^-1^) + urea N (30 kg ha^-1^) as a base + urea N (30 kg ha^-1^) topdressing at panicle initiation stage; N_5_: controlled-release N (60 kg ha^-1^) + urea N (60 kg ha^-1^) as a base + urea N (30 kg ha^-1^) topdressing at panicle initiation stage; N_6_: controlled-release N (30 kg ha^-1^) + urea N (90 kg ha^-1^) as a base + urea N (30 kg ha^-1^) topdressing at panicle initiation stage.

Field experiments were conducted in Chongzhou, Sichuan Province, China (103°38′E, 30°33′N) (**Figure 1A**), in 2017 and 2018. The study site has a subtropical monsoon humid climate, and the rainfall, average temperature, and sunshine hours during the rice growing season (April to September) were 881.50 mm, 22.56°C, and 761.10 h in 2017 and 916.60 mm, 22.93°C, and 755.80 h in 2018, respectively. Yixiangyou 2115, a representative hybrid *indica* rice cultivar bred by Sichuan Agricultural University that is widely planted in South China, was used. It has high grain yield, NUE, and pest-resistance characteristics and presents a growth period of 148-153 days from sowing to maturity. Before rice was cultivated, wheat was planted in the experimental fields. The soil was sandy loam (0~20 cm) before the initiation of the experiments. The characteristics in 2017 were as follows: 23.01 g kg^-1^ organic matter, 1.70 g kg^-1^ total N, 102.14 mg kg^-1^ available N, 59.01 mg kg^-1^ available P, and 104.12 mg kg^-1^ available K. The characteristics in 2018 were as follows: 23.96 g kg^-1^ organic matter, 1.74 g kg^-1^ total N, 107.20 available N, 58.43 mg kg^-1^ available P, and 99.85 mg kg^-1^ available K. Conventional urea fertilizer (46% N) and polymer-coated controlled-release urea fertilizer (44% N; Shandong Kingenta Ecological Engineering Co., Ltd.) were used as the N fertilizers. The N-release characteristics of the polymer-coated controlled-release N shown in **Figure 1B** were determined before the experiment using the water extraction method (Tomaszewska and Jarosiewicz, 2002). The cumulative release rate of N reached 80% within 55 d (**Figure 1B**). In addition, phosphorus (P) (calcium superphosphate, 12% P_2_O_5_) and potassium (K) (potassium chloride, 60% K_2_O) fertilizers were also used. Rice seedlings were raised in pots in blanket-shaped seedling trays (China National Rice Research Institute) and transplanted using a Kubota SPU-48C transplanter (Kubota Agricultural Machinery Suzhou Co., Ltd.).

### 2.2 Experimental design and field management

A one-factor field experiment with seven N treatments was conducted in 2017 and 2018 using a randomized complete block design (**Figure 1C**). The N treatments (**Table 1**) were as follows: (1) no N application (N_0_); (2) local conventional urea-N (180 kg ha^-1^) practice (N_1_, 120 kg N ha^-1^ as a base + 60 kg N·ha^-1^ as a tiller fertilizer) (Sun et al., 2012); and (3) application of polymer-coated controlled-release N fertilizer (150 kg ha^-1^) under total N reduction (N_2_, 100% as a base). Previous studies by our group and others have shown that most hybrid *indica* rice belongs to heavy-panicle type varieties (single panicle weight > 5.0 g) and revealed that topdressing conventional urea-N (panicle N application accounts for 20% of the total N amount) at the panicle initiation stage (Yang et al., 2014; Sun et al., 2023a) was beneficial for improving grain-filling and grain yield. Based on these, in the present study, we used 150 kg N ha^-1^ as a base level and conventional urea-N (30 kg ha^-1^) at 20% of the total N amount as a top dressing; four treatments with different controlled-release N and conventional urea-N ratios were established: (4) 80%:0% (N_3_); (5) 60%:20% (N_4_); (6) 40%:40% (N_5_); and (7) 20%:60% (N_6_). The cumulative amounts of N released by the different controlled-release N fertilizer treatments are shown in **Figure 1D**. For each plot, P and K were applied as basal fertilizers at rates of 75 kg ha^-1^ P_2_O_5_ and 150 kg ha^-1^ K_2_O, respectively. Rice seeds were sown on April 15 in both years using a dry rice nursery and 75 g of seeds (dry seeds) in each bowl blanket tray. The unit plot size was 54.0 m^2^ (9.0 m length and 6.0 m width). The row spacing and plant spacing were 30 cm and 18 cm, respectively, with the seedlings sown using an SPU-48C rice transplanter in triplicate for each treatment on May 10, 2017, and May 13, 2018. Ridges (40 cm wide and 30 cm high) were built between plots and then covered with a plastic film to avoid the overflow of water and fertilizer. The high-efficiency irrigation technique described by Sun et al. (2012) was implemented in each plot. Chemical pesticides were used to avoid yield losses and experimental errors caused by insects, diseases, and weeds.

**Table 1:** The treatments of controlled-release N combined with conventional urea N management (kg ha^-1^).

Treatments	Total N amount	Basal N fertilizer (1d before transplanting)		N topdressing of conventional urea	
		Controlled-release N	Conventional urea N	Tiller fertilizer (7d after transplanting)	Panicle fertilizer (4^th^ leaves emerged from the top)
N_0_	0	0	0	0	0
N_1_	180	0	120	60	0
N_2_	150	150	0	0	0
N_3_	150	120	0	0	30
N_4_	150	90	30	0	30
N_5_	150	60	60	0	30
N_6_	150	30	90	0	30

N_0_: no N application; N_1_: farmer urea N (180 kg ha^-1^) practice; N_2_: controlled-release N (150 kg ha^-1^) as a base; N_3_: controlled-release N (120 kg ha^-1^) as a base + urea N (30 kg ha^-1^) topdressing at panicle initiation stage; N_4_: controlled-release N (90 kg ha^-1^)+ urea N (30 kg ha^-1^) as a base + urea N (30 kg ha^-1^) topdressing at panicle initiation stage; N_5_: controlled-release N (60 kg ha^-1^)+ urea N (60 kg ha^-1^) as a base + urea N (30 kg ha^-1^) topdressing at panicle initiation stage; N_6_: controlled-release N (30 kg ha^-1^)+ urea N (90 kg ha^-1^) as a base + urea N (30 kg ha^-1^) topdressing at panicle initiation stage.

### 2.3 Measurement terms and methods

#### 2.3.1 Leaf area

0.27 m^2^ plants in each plot were sampled at the jointing and full-heading stages in 2017 and 2018. The samples were divided into the top three leaves (highly effective leaves), other leaves, stem sheaths, and panicles (full-heading stage). The area of the highly effective leaves and other leaves was measured using a CID-203 leaf area analyzer (CID Company, USA). The leaf area index (LAI, a multiple of the total area of plant leaves per unit land area) at the jointing and full-heading stages and high effective LAI at the full-heading stage was calculated following Lyu et al. (2021a). Then, the stem sheaths, leaves, and panicles at each growth stage were exposed to 105 °C for 40 min and dried at 80 °C to a constant weight.

#### 2.3.2 Net photosynthetic rate of flag leaf and root activity

A total of 0.27 m^2^ plants in each plot were sampled at 0, 15, and 30 d after the full-heading stage in 2017 and 2018. According to the methods described by Sun et al. (2012), the net photosynthetic rate of flag leaves of the main stem was measured using a Li-6400 photosynthetic instrument (Li-COR Inc., NE, USA) from 9:30 to 11:00 am when the photosynthetic active radiation above the canopy was 1200 mmol·m^−2^·s^−1^. Measurements were repeated three times for each leaf, and the average value was recorded.

Root activity was determined at the aforementioned stages. The roots in the soil were dug out using a spade (the soil volume around the roots was 30×18×20 cm), carefully rinsed, and detached from their nodal bases. Root activity was measured according to the method of Ramasamy et al. (1997); 2.0 g of fresh roots was transferred into a 100 mL flask containing 25.0 mL of 50.0 mg·L^-1^α-NA and 25.0 mL phosphate buffer (0.1 mol·L^-1^, pH 7.0), The flasks were incubated for 2 h at 25 °C in an end-over-end shaker. After incubation, the aliquots were filtered, and 2.0 mL was mixed with 1.0 mL NaNO_2_ (100.0 μg·mL^-1^) and 1.0 mL sulphanilic acid (1%) and the resulting color was measured at 510 nm using a spectrophotometer (Shimadzu-1700, Japan), and expressed as μg α-NA g^-1^·DW·h^-1^.

#### 2.3.3 Grain-filling

Panicles with relatively consistent flowering times and sizes were selected at the heading stage. Two hundred panicles were marked with a tag in each plot, and ten tagged panicles were collected twice (on the 3rd and 7th days, every 7 days) at 7 d intervals from flowering to maturity (Sun et al., 2018). During each sampling period, the superior and inferior grains were dried, threshed, and weighed. Superior and inferior grains were classified as follows: those on three primary branches at the panicle base, except for the 2nd grain from the top, were considered superior grains, while those on the secondary branches of three primary branches at the panicle base, except for the 1st grain from the top, were considered inferior grains. Richard’s equation was used for fitting according to the methods described by Yang et al. (2003); Jiang et al. (2016), and Sun et al. (2018):


(1) $$W=A/{\left(1+Be^{-kt}\right)}^{1/N}$$

(2) $$G=AkBe^{-kt}/N{\left(1+Be\right)}^{(N+1)/N}$$

where *W* is the grain weight (mg); *A* is the final grain weight; *t* is the time after flowering (d); *B, k*, and *N* are the equation parameters; and *G* is the grain-filling rate (mg·kernel^-1^·d^-1^).

#### 2.3.4 Nitrogen content

A total of 0.27 m^2^ plants in each plot were sampled at the full-heading and maturity stages. The plant samples were divided into three parts (leaves, stem sheath, and panicle), dried to a constant weight at 80°C, and then weighed. The samples were milled and passed through a 0.5 mm sieve. After digestion in concentrated H_2_SO_4_ with a fixed N catalyst, an automatic Kjeldahl apparatus (FOSS-8400, Sweden) was used to determine the N content according to the Kjeldahl-N method (Yoshida et al., 1976).

#### 2.3.5 Yield and yield components

The effective panicle number was determined from 5.0 m^2^ in each plot at the maturity stage. 0.54 m^2^ plants in each plot were sampled from each plot to measure the yield components. The panicles were hand-threshed, unfilled spikelets were separated from filled spikelets by winnowing (CFY-II, Top Instrument Co., Ltd., Zhejiang, China), and the 1000-grain weight and filled grain percentage were calculated. The grain yield was determined by harvesting each 18.0 m^2^ plot without border plants and adjusting it to a standard moisture content of 13.5% using a grain moisture meter (PM-8188-A, Kett Electric Laboratory, Tokyo, Japan).

### 2.4 Indicator calculation

#### 2.4.1 Population photosynthetic potential and population growth rate

The population photosynthetic potential and population growth rate were calculated according to the methods of Li et al. (2011):


(3) $$Population\;photosynthetic\;potential\;{\text{(m}}^{2}{\cdot \text{d}\cdot \text{hm}}^{-2}\text{)=}\frac{1}{2}({\text{L}}_{1}{\text{+L}}_{2})\times ({\text{t}}_{2}{\text{-t}}_{1})$$

(4) $$Population\;growth\;rate\;{(\text{g·m}}^{-2}{\text{·d}}^{-1}\text{)=}({\text{W}}_{2}{\text{-W}}_{1})/({\text{t}}_{2}{\text{-t}}_{1})$$

where L_1_ and L_2_ are the leaf areas at the jointing and full-heading stages, respectively; W_1_ and W_2_ are the dry matter weights at the jointing and full-heading stages, respectively; and t_1_ and t_2_ are the times at the jointing and full-heading stages, respectively.

#### 2.4.2 Nitrogen utilization

The N transport amount (NT), N transport efficiency (NTE), N transport contribution rate (NTEC), N harvest index (NHI), N biomass production efficiency (NBPE), N grain production efficiency (NGPE), N agronomic efficiency (NAE), N partial factor productivity (NPP), and N recovery efficiency (NRE) were calculated according to the methods described by Sun et al. (2020b) and Ma et al. (2020).


(5) $${\text{NT (kg·hm}}^{-2}\text{)=}\left(\begin{array}{c} \text{N accumulation in stem}\;shea\text{ths or leaves at full-heading }\\ \text{-N accumulation in stem}\;shea\text{ths or leaves at maturity } \end{array}\right)$$

(6) NTE (%)=NT in stem sheaths or leaves/N accumulation in stem sheaths or leaves×100

(7) NTEC (%)=NT in stem sheaths or leaves/N accumulation in panicle from full-heading to maturity×100

(8) NHI (%)=N accumulation in grains at maturity/total N accumulation at maturity×100

(9) $${\text{NBPE (kg·kg}}^{-1}\text{)=biomass accumulation at maturity}/\text{total N accumulation at maturity}$$

(10) $${\text{NGPE (kg·kg}}^{-1}\text{)=grain yield}/\text{total N accumulation at maturity}$$

(11) $$\text{NAE}\;{\text{(kg·kg}}^{-1}\text{)=}\left(\text{grain yield in N supply - grain yield in zero N supply}\right)/\text{N supply rate}$$

(12) $${\text{NPP (kg·kg}}^{-1}\text{)=grain yield in N supply}/\text{N supply rate}\;$$

(13) $$\text{NRE }\left(\%\right)=\left(\begin{array}{c} \text{total N accumulation in N supply at maturity }\\ \text{-total N accumulation in zero N supply at maturity} \end{array}\right)/\text{N supply rate×}100$$

#### 2.4.3 Grain-filling characteristics

The initial growth potential of the grain (R_0_), time of maximum growth rate (T_max_), maximum filling rate (G_max_), mean filling rate during the filling stage (G_mean_), and grain-filling accumulation were calculated based on the three phases (*T*_1_, *T*_2_, and *T*_99_) after flowering, which are introduced in equation (1). The mean grain-filling rate (MGR) of the three filling periods 0-T_1_ (early-filling), T_1_-T_2_ (middle-filling), and T_2_-T_99_ (last-filling) was calculated based on the three phases, filling material accumulation, and the ratio of grain-filling contributing to *A* value (RGC) in each phase was calculated according to the methods of Sun et al. (2020b):


(14) $$R_{0}=k/N$$

(15) $$T_{max}{=}^{(1nB-1nN)/k}$$

(16) $${\text{G}}_{\text{max }}{\text{=AkBe}}^{{\text{-kT}}_{\text{max}}}/\text{N}{\left({1+Be}^{{\text{-kT}}_{\text{max}}}\right)}^{\left(N+1\right)/\text{N}}\;$$

(17) $$G_{mean}=Ak/2\left(N+2\right)$$

(18) $$T_{1}\text{= -ln[(}N^{2}+3N+N\cdot \sqrt{N^{2}+6N+5})/2B\text{]/}k$$

(19) $$T_{2}=-1n[(N^{2}+3N-N\cdot \sqrt{N^{2}+6N+5)}/2B]/k$$

(20) $$T_{99}{\text{= -ln\{[(100/99)}}^{N}-1]/B\}/k$$

### 2.5 Statistical analysis

Data analysis and graphing were performed using SAS 8.1 (SAS Institute, Cary, NC, USA) and SigmaPlot 10.0 (Systat Software Inc., Chicago, IL, USA), respectively. The treatment means were tested by the least significant difference (LSD; *P<* 0.05). Structural equation modeling (SEM) was conducted using Amos 24.0 to fit the plot (Livote and Wyka, 2008). Principal component analysis was completed with Origin 2023 (OriginLab Corp., Northampton, MA).

## 3 Results

### 3.1 Yield and yield components

The effects of N fertilizer management on grain yield and its components in machine-transplanted rice in both years were significant (**Table 2**). The N application treatment increased the number of effective panicles and filled spikelets and significantly increased the grain yield. Compared with the conventional urea application (N_1_) treatment, all treatments with N fertilizer reduction via controlled-release N fertilizer combinations (N_2_, N_3_, N_4_, N_5_, and N_6_) significantly increased the grain yield by 6.08-25.75% except for the N_6_ treatment in 2018. Compared to the N_2_ treatment, the grain yield and its components initially increased and then decreased significantly in the N_3_, N_4_, N_5_, and N_6_ treatments (with 20% conventional urea-N panicle fertilizer) as the proportion of controlled-release N fertilizer in base fertilizer decreased. The N_4_ treatment included a ratio of controlled-release N fertilizer to conventional urea-N of 60%:20% (one-time base fertilizer application and 20% conventional urea-N panicle fertilizer topdressing), and it could significantly increase the effective panicles 3.30-8.89% and filled grains 1.84-6.71% and further significantly increase the grain yield 5.03-21.69%. The grain yield of the N_4_ treatment was the highest in both years and represented the optimal controlled-release N fertilizer and conventional urea-N fertilizer application optimization model in this study. This indicates that the application of controlled-release N fertilizer can reduce the total input of conventional N fertilizer, and the appropriate combination of controlled-release N and available N fertilizer can further optimize the yield components and achieve a high yield. In the present study, the N_4_ treatment was the most suitable under the 150 kg hm^-2^ N application rate.

**Table 2:** Effects of methodical controlled-release N combined with conventional urea management on yield and its components of machine-transplanted hybrid *indica* rice.

Year	N treatments	Grain yield (kg·hm^-2^)	Effective panicles (×10^4^·hm^-2^)	Filled spikelets (No. panicle^-1^)	Filled grains (%)	1000-grain weight (g)
2017	N_0_	8596.8 d	187.2 d	137.2 d	90.98 a	34.13 b
	N_1_	9999.2 c	217.1 c	143.0 cd	83.96 e	32.76 c
	N_2_	10961.3 b	223.6 bc	146.2 c	84.86 cd	34.11 b
	N_3_	11808.2 a	227.4 b	155.6 ab	85.75 c	34.24 ab
	N_4_	12401.6 a	234.9 a	157.2 a	86.87 b	34.85 a
	N_5_	10979.6 b	220.0 bc	150.2 b	84.03 de	34.45 ab
	N_6_	10607.0 b	217.0 c	147.6 c	83.27 e	34.14 b
	*F* value	4.28*	7.20**	7.88**	11.82**	4.02*
2018	N_0_	8457.7 f	188.9 d	135.2 d	93.77 a	33.76 ab
	N_1_	9961.0 e	211.5 c	147.1 c	86.27 ef	32.61 c
	N_2_	10698.8 cd	219.2 bc	150.4 c	88.14 d	32.71 c
	N_3_	11458.6 b	220.3 b	156.8 ab	90.16 c	34.36 a
	N_4_	12526.3 a	233.9 a	161.5 a	91.82 b	33.43 b
	N_5_	10997.2 bc	218.3 bc	152.9 bc	87.57 de	33.21 bc
	N_6_	10293.3 de	214.8 bc	150.5 c	86.05 f	32.85 c
	*F* value	9.86**	8.25**	7.21**	14.70**	8.48**

N_0_: no N application; N_1_: farmer urea N (180 kg ha^-1^) practice; N_2_: controlled-release N (150 kg ha^-1^) as a base; N_3_: controlled-release N (120 kg ha^-1^) as a base + urea N (30 kg ha^-1^) topdressing at panicle initiation stage; N_4_: controlled-release N (90 kg ha^-1^)+ urea N (30 kg ha^-1^) as a base + urea N (30 kg ha^-1^) topdressing at panicle initiation stage; N_5_: controlled-release N (60 kg ha^-1^)+ urea N (60 kg ha^-1^) as a base + urea N (30 kg ha^-1^) topdressing at panicle initiation stage; N_6_: controlled-release N (30 kg ha^-1^)+ urea N (90 kg ha^-1^) as a base + urea N (30 kg ha^-1^) topdressing at panicle initiation stage. *, P < 0.05; **, P < 0.01. Different lowercase letters indicate significant (P < 0.05) differences among treatments under the same year.

### 3.2 Photosynthetic production

N fertilizer management has a significant impact on the LAI at the jointing and full-heading stages, high-efficiency LAI, high-efficiency leaf area rate at the full-heading stage, and population photosynthetic potential and growth rate from the jointing to the full-heading stage of machine-transplanted rice (**Table 3**). Except for the LAI at the jointing stage of N_2_ treatment in 2018, which was not significantly reduced, the LAI at the jointing stage was significantly reduced by 5.31-12.56% under the N_2_, N_3_, N_4_, N_5_, and N_6_ treatments compared with that in the N_1_ treatment. Compared with the N_1_ treatment, the N_2_ and N_3_ treatments could also compensate for the gap between the LAI at the full-heading stage and the N_1_ treatment, improve the efficient LAI at the full-heading stage to varying degrees, and significantly increase the efficient leaf area rate 1.92-7.81%. Although the population photosynthetic potential of the N_2_ treatment from the jointing to the full-heading stage was lower than that of the N_1_ treatment in both years, the population growth rate of the N_2_ and N_3_ treatments from the jointing to the full-heading stage was significantly higher 12.09-26.83% than that of the N_1_ treatment. In addition, compared to the N_3_ treatment, the LAI at the full-heading stage, high-efficiency LAI and high-efficiency LAI rate at the full-heading stage, population photosynthetic potential, and growth rate from the jointing to full-heading stage increased and then decreased significantly in the N_3_, N_4_, N_5_, and N_6_ treatments with a decrease of the proportion of controlled-release N fertilizer in base fertilizer, with the greatest values observed in the N_4_ treatment. This indicates that the N_4_ treatment can further improve the high-efficiency LAI 8.44-36.01% and high-efficiency LAI rate 1.67-9.41% at the full-heading stage, increase the photosynthetic potential 4.81-21.58% and population growth rate 5.32-28.47% from the jointing to the full-heading stage, and guarantee high yields (**Table 2**).

**Table 3:** Effects of methodical controlled-release N combined with conventional urea management on photosynthetic production of machine-transplanted hybrid *indica* rice.

Year	N treatments	LAI		High effective LAI at full-heading stage	High effective LAI rate of full-heading stage (%)	Photosynthetic potential from jointing to full-heading stage (×10^4^ m^2^·d·hm^-2^)	Population growth rate from jointing to full-heading stage (g·m^-2^·d^-1^)
		Jointing stage	Full-heading stage				
2017	N_0_	2.47 e	3.24 e	2.04 d	62.96 d	91.36 d	19.44 d
	N_1_	4.38 a	5.13 b	3.48 bc	67.84 c	152.16 b	19.72 cd
	N_2_	4.11 b	4.69 bcd	3.38 bc	72.07 b	145.20 c	22.18 b
	N_3_	3.83 d	4.98 bc	3.67 b	73.69 b	154.18 b	25.01 a
	N_4_	4.03 bc	6.13 a	4.64 a	75.69 a	172.38 a	26.02 a
	N_5_	4.06 bc	4.52 cd	3.11 c	68.81 c	145.86 c	23.02 b
	N_6_	3.90 cd	4.44 d	3.04 c	68.47 c	141.78 c	20.66 c
	*F* value	8.82**	19.33**	51.92**	25.02**	8.91**	4.90*
2018	N_0_	2.42 c	3.23 d	2.06 e	63.78 d	90.40 d	19.63 e
	N_1_	4.33 a	5.17 bc	3.46 c	66.92 c	151.92 b	20.09 de
	N_2_	4.21 ab	4.91 cd	3.38 cd	68.84 b	150.48 b	22.52 c
	N_3_	4.04 b	5.57 ab	4.16 b	74.73 a	168.00 a	25.19 b
	N_4_	4.10 b	6.06 a	4.63 a	76.40 a	172.72 a	26.53 a
	N_5_	4.12 b	4.67 d	3.15 cd	67.45 bc	149.43 bc	22.57 c
	N_6_	4.02 b	4.49 d	3.02 d	67.26 c	144.67 c	20.65 d
	*F* value	7.06**	20.04**	60.90**	31.63**	9.88**	4.01*

N_0_: no N application; N_1_: farmer urea N (180 kg ha^-1^) practice; N_2_: controlled-release N (150 kg ha^-1^) as a base; N_3_: controlled-release N (120 kg ha^-1^) as a base + urea N (30 kg ha^-1^) topdressing at panicle initiation stage; N_4_: controlled-release N (90 kg ha^-1^)+ urea N (30 kg ha^-1^) as a base + urea N (30 kg ha^-1^) topdressing at panicle initiation stage; N_5_: controlled-release N (60 kg ha^-1^)+ urea N (60 kg ha^-1^) as a base + urea N (30 kg ha^-1^) topdressing at panicle initiation stage; N_6_: controlled-release N (30 kg ha^-1^)+ urea N (90 kg ha^-1^) as a base + urea N (30 kg ha^-1^) topdressing at panicle initiation stage. LAI: leaf area index. *, P < 0.05; **, P < 0.01. Different lowercase letters indicate significant (P < 0.05) differences among treatments under the same year.

### 3.3 Net photosynthetic rate and root activity

**Figure 2:**
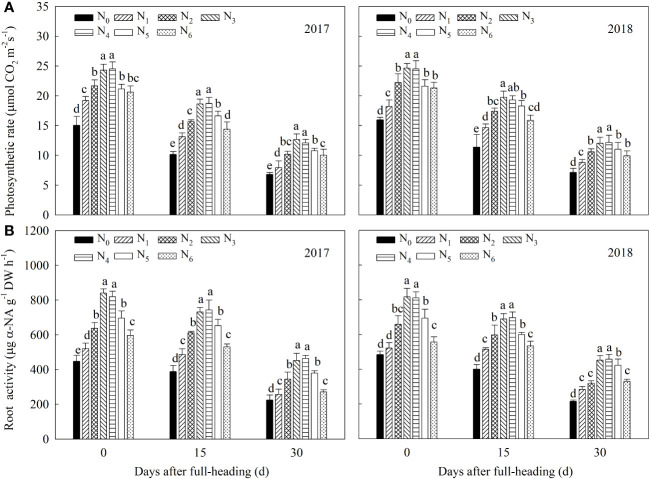
Effects of methodical controlled-release N combined with conventional urea management on photosynthetic rate **(A)** and root activity **(B)** during filling stage of machine-transplanted hybrid *indica* rice. N_0_: no N application; N_1_: farmer urea N (180 kg ha^-1^) practice; N_2_: controlled-release N (150 kg ha^-1^) as a base; N_3_: controlled-release N (120 kg ha^-1^) as a base + urea N (30 kg ha^-1^) topdressing at panicle initiation stage; N_4_: controlled-release N (90 kg ha^-1^)+ urea N (30 kg ha^-1^) as a base + urea N (30 kg ha^-1^) topdressing at panicle initiation stage; N_5_: controlled-release N (60 kg ha^-1^) + urea N (60 kg ha^-1^) as a base + urea N (30 kg ha^-1^) topdressing at panicle initiation stage; N_6_: controlled-release N (30 kg ha^-1^) + urea N (90 kg ha^-1^) as a base + urea N (30 kg ha^-1^) topdressing at panicle initiation stage. Different letters from top the column indicate statistical significance (*P*< 0.05) differences among treatments within the same period under the same year.

As shown in **Figure 2**, N fertilizer management significantly increased the flag leaf net photosynthetic rate (*P*_n_) (**Figure 2A**) and root activity (**Figure 2B**) after full-heading, with these parameters decreasing with the growth process. At the same growth stage, the combined application of reduced N fertilizer and controlled-release N fertilizer (N_2_, N_3_, N_4_, N_5_, and N_6_) showed different degrees of increase in the *P*_n_ 7.22-31.12% and root activity 5.09-54.58% compared with the N_1_ treatment. In the N_3_, N_4_, N_5_, and N_6_ treatments, as the proportion of controlled-release N fertilizer in the base fertilizer decreased, the *P*_n_ and root activity initially increased and then decreased significantly at each growth stage compared with that of the N_2_ treatment. In addition, although the advantages of N_3_ and N_4_ were obvious, the difference was not significant in both years. Increasing the proportion of available N fertilizer in the base fertilizer based on the N_4_ treatment would lead to a significant decrease in *P*_n_ 8.09-20.34% and root activity 12.93-34.72% in the N_5_ and N_6_ treatments at different growth stages after full-heading.

### 3.4 Grain-filling characteristics

#### 3.4.1 Dynamics of increasing grain weight and grain-filling rate

**Figure 3:**
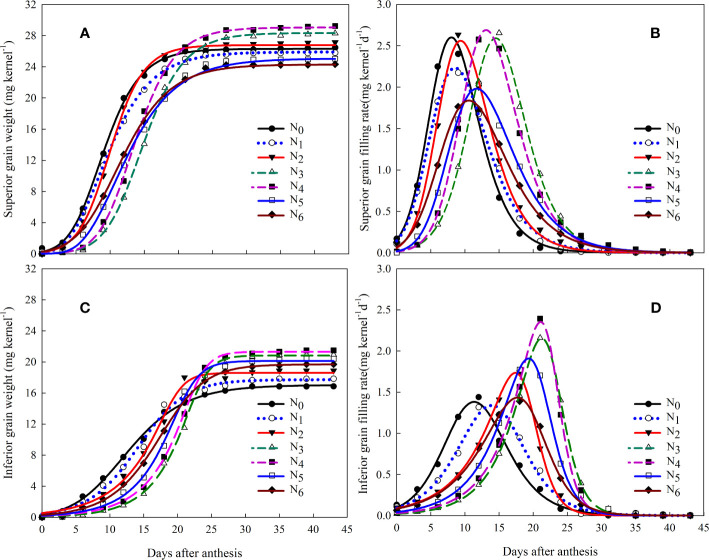
Effects of methodical controlled-release N combined with conventional urea management on grain-filling process **(A)** and rate **(B)** in inferior grains process **(C)** and rate **(D)** in superior grains of machine-transplanted hybrid *indica* rice. N_0_: no N application; N_1_: farmer urea N (180 kg ha^-1^) practice; N_2_: controlled-release N (150 kg ha^-1^) as a base; N_3_: controlled-release N (120 kg ha^-1^) as a base + urea N (30 kg ha^-1^) topdressing at panicle initiation stage; N_4_: controlled-release N (90 kg ha^-1^) + urea N (30 kg ha^-1^) as a base + urea N (30 kg ha^-1^) topdressing at panicle initiation stage; N_5_: controlled-release N (60 kg ha^-1^) + urea N (60 kg ha^-1^) as a base + urea N (30 kg ha^-1^) topdressing at panicle initiation stage; N_6_: controlled-release N (30 kg ha^-1^)+ urea N (90 kg ha^-1^) as a base + urea N (30 kg ha^-1^) topdressing at panicle initiation stage.

Under different N fertilizer management, the dynamic changes in the weight gain of superior and inferior grains after anthesis of mechanically transplanted rice were consistent with that of the Richards model (**Figure 3**). From the parameters of grain-filling (**Table 4**), the initial growth potential (R_0_) of superior and inferior grains was the highest in the N_0_ treatment, followed by the N_1_ treatment. The effects of the combined application of N fertilizer reduction and controlled-release N fertilizer (N_2_, N_3_, N_4_, N_5_, and N_6_) on the R_0_ of the superior and inferior grains were different. With a decrease in the proportion of controlled-release N fertilizer in the base fertilizer, the R_0_ of superior grains initially decreased and then increased, with the lowest observed in the N_3_ treatment; moreover, the inferior grain R_0_ initially increased and then decreased, with the highest in the N_3_ treatment. The time to reach the maximum growth rate (T_max_) of the superior and inferior grains after anthesis was N_3_>N_4_>N_5_>N_6_>N_2_>N_1_>N_0_, indicating that R_0_ would advance T_max_ while the application of 20% conventional urea panicle fertilizer (N_3_, N_4_, N_5_, N_6_) would delay T_max_. With an increase in the proportion of controlled-release N fertilizer in the base fertilizer (N_3_ and N_4_ treatments), T_max_ was further delayed, which indirectly demonstrated that there was a significant inter-regulation effect between conventional urea management and controlled-release N fertilizer treatments. Consistent trends of the maximum filling rate (G_max_) and mean filling rate during the filling period (G_mean_) under different N fertilizer management regimes showed that the G_max_ and G_mean_ of the N_4_ treatment were the best, followed by the N_3_ treatment. This indicates that although the N_3_ and N_4_ treatments had lower R_0_ and delayed T_max_ compared to the other treatments, they had higher G_max_ and G_mean_ values and higher filling intensity. However, compared to the N_3_ treatment, the N_4_ treatment could optimize the grain-filling parameters, advance the T_max_, and further improve the G_max_ and G_mean_.

**Table 4:** Effects of methodical controlled-release N combined with conventional urea management on grain filling parameters of machine-transplanted hybrid *indica* rice.

Grain position	N treatments	R_0_	T_max_ (d)	G_max_ (mg·kernel^-1^·d^-1^)	G_mean_ (mg·kernel^-1^·d^-1^)
Superior grain	N_0_	0.881	8.26	2.63	1.78
	N_1_	0.879	8.29	2.21	1.50
	N_2_	0.877	9.18	2.54	1.72
	N_3_	0.481	14.50	2.59	1.74
	N_4_	0.696	13.09	2.69	1.82
	N_5_	0.856	10.49	1.92	1.30
	N_6_	0.861	10.24	1.82	1.24
Inferior grain	N_0_	0.377	12.17	1.29	0.86
	N_1_	0.283	14.04	1.35	0.90
	N_2_	0.192	17.39	1.73	1.10
	N_3_	0.245	21.26	2.16	1.39
	N_4_	0.226	21.09	2.35	1.49
	N_5_	0.221	19.34	1.91	1.23
	N_6_	0.193	17.69	1.43	0.93

N_0_: no N application; N_1_: farmer urea N (180 kg ha^-1^) practice; N_2_: controlled-release N (150 kg ha^-1^) as a base; N_3_: controlled-release N (120 kg ha^-1^) as a base + urea N (30 kg ha^-1^) topdressing at panicle initiation stage; N_4_: controlled-release N (90 kg ha^-1^)+ urea N (30 kg ha^-1^) as a base + urea N (30 kg ha^-1^) topdressing at panicle initiation stage; N_5_: controlled-release N (60 kg ha^-1^)+ urea N (60 kg ha^-1^) as a base + urea N (30 kg ha^-1^) topdressing at panicle initiation stage; N_6_: controlled-release N (30 kg ha^-1^)+ urea N (90 kg ha^-1^) as a base + urea N (30 kg ha^-1^) topdressing at panicle initiation stage. R_0_: starting growth potential of grain; T_max_: time of maximum growth rate; G_max_: maximum filling rate during filling stage; G_mean_: mean filling rate during filling stage.

#### 3.4.2 Early, middle, and late filling stage grain-filling characteristics

From the early grain-filling characteristics of machine-transplanted rice (**Table 5**), the filling days of the superior and inferior grains in each combination of N fertilizer reduction and controlled-release N fertilizer (N_2_, N_3_, N_4_, N_5_, and N_6_) treatments were significantly increased compared with the N_0_ and N_1_ treatments. The MGR of the superior grains decreased, whereas the MGR of the inferior grains increased to different degrees. The contribution rate of grain-filling to the *A* value (RGC) was related to the number of days of grain-filling and MGR. In the early-filling stage, the superior grains of the RGC were the highest in the N_3_ treatment, followed by the N_4_ treatment, and the inferior grains were the highest in the N_4_ treatment, followed by the N_3_ treatment. It can be seen from **Table 5** that the MGR was the highest in the middle-filling stage. Although the duration of the grain-filling period was short, the RGC of the superior and inferior grains was more than 50%. Compared with the N_1_ treatment, the MGR of the inferior grains increased significantly by 4.62-47.19% with the N_2_, N_3_, N_4_, N_5_, and N_6_ treatments. Compared with the N_2_ treatment, under the N_3_, N_4_, N_5_, and N_6_ treatments, with a decrease in the proportion of controlled-release N fertilizer in the base fertilizer, the MGR of the superior and inferior grains initially increased and then decreased significantly, with the highest values observed in the N_4_ treatment. Under each N treatment at the late filling stage, the superior and inferior grains were the highest in the N_4_ treatment, followed by the N_3_ treatment. The RGC of the N_3_ and N_4_ treatments was higher in the early and middle-filling stages. In addition, the number of filling days in the late stage decreased, and its contribution rate to grains also decreased.

**Table 5:** Effects of methodical controlled-release N combined with conventional urea management on grain filling characteristics at the early filling, middle filling, and late filling stage of machine-transplanted hybrid *indica* rice.

Grain position	N treatments	Early filling stage			Middle filling stage			Late filling stage		
		Days (d)	MGR (mg·kernel^-1^·d^-1^)	RGC %	Days (d)	MGR (mg·kernel^-1^·d^-1^)	RGC %	Days (d)	MGR (mg·kernel^-1^·d^-1^)	RGC %
Superior grain	N_0_	4.90	0.683	14.54	6.71	2.289	63.26	10.48	0.622	21.20
	N_1_	4.35	0.704	12.23	7.87	1.921	60.45	12.65	0.521	26.31
	N_2_	5.59	0.599	12.76	7.18	2.207	60.35	11.43	0.599	25.89
	N_3_	10.74	0.460	17.16	7.53	2.263	59.16	10.46	0.624	22.67
	N_4_	9.29	0.443	14.87	7.81	2.340	60.30	11.34	0.638	23.84
	N_5_	5.94	0.492	11.65	9.11	1.668	60.56	14.90	0.451	26.79
	N_6_	5.62	0.496	11.56	9.24	1.581	60.58	15.15	0.428	26.86
Inferior grain	N_0_	7.69	0.399	18.90	8.97	1.126	64.92	12.20	0.312	15.18
	N_1_	9.79	0.407	22.49	8.51	1.189	57.18	10.21	0.335	19.33
	N_2_	14.42	0.502	35.41	5.94	1.540	50.08	4.65	0.468	13.51
	N_3_	18.42	0.417	35.48	5.68	1.907	51.7 1	5.08	0.564	12.71
	N_4_	18.55	0.450	36.47	5.08	2.089	50.86	4.10	0.634	11.67
	N_5_	16.26	0.423	34.01	6.16	1.690	51.47	5.46	0.501	13.52
	N_6_	13.79	0.429	31.61	7.80	1.267	52.79	7.35	0.372	14.60

N_0_: no N application; N_1_: farmer urea N (180 kg ha^-1^) practice; N_2_: controlled-release N (150 kg ha^-1^) as a base; N_3_: controlled-release N (120 kg ha^-1^) as a base + urea N (30 kg ha^-1^) topdressing at panicle initiation stage; N_4_: controlled-release N (90 kg ha^-1^)+ urea N (30 kg ha^-1^) as a base + urea N (30 kg ha^-1^) topdressing at panicle initiation stage; N_5_: controlled-release N (60 kg ha^-1^)+ urea N (60 kg ha^-1^) as a base + urea N (30 kg ha^-1^) topdressing at panicle initiation stage; N_6_: controlled-release N (30 kg ha^-1^)+ urea N (90 kg ha^-1^) as a base + urea N (30 kg ha^-1^) topdressing at panicle initiation stage. MGR, Mean grain filling rate at the certain reproductive stage; RGC, Ratio of the grain filling contributed to the final grain weight.

### 3.5 Nitrogen utilization characteristics

#### 3.5.1 Nitrogen transport in stem sheaths and leaves

N management in both years had a significant effect on the NT, NTE, and NTEC of stem sheaths (leaves) from the full-heading to maturity stages (**Table 6**). Compared to the N_1_ treatment, the N_2_ treatment showed significant increases in the NT, NTE, and NTEC of the stems sheaths by 5.00-21.98% in the two-year experiments and the NT, NTE, and NTEC of the leaves by 7.01-17.32% in 2018 from full-heading to maturity stage; however, such changes were not observed for the NT, NTE, and NTEC of the leaves in 2017. Compared to the N_2_ treatment, significant increases in the NT, NTE, and NTEC of the stem sheaths (leaves) from full-heading to maturity stage, and then a significant decrease was observed in the N_3_, N_4_, N_5_, and N_6_ treatments as the proportion of controlled-release N fertilizer in the base fertilizer decreased, with the greatest changes observed in the N_4_ treatment. This finding further confirms that the optimal combination of controlled-release N fertilizer and available N fertilizer improved the transport of N from the stem sheaths (leaves) to panicles during the grain-filling stage.

**Table 6:** Effects of methodical controlled-release N combined with conventional urea management on N translocation in stem-sheaths and leaves from full-heading to maturity stage of machine-transplanted hybrid *indica* rice.

Year	N treatments	Stem-sheaths			Leaves		
		NT (kg·hm^−2^)	NTE (%)	NTEC (%)	NT (kg·hm^−2^)	NTE (%)	NTEC (%)
2017	N_0_	11.76 d	38.29 e	23.24 c	20.27 e	63.58 a	40.08 a
	N_1_	18.46 c	41.10 d	22.99 c	27.07 cd	54.41 c	33.71 d
	N_2_	21.32 b	43.14 bc	25.57 b	28.49 c	55.26 c	34.17 cd
	N_3_	23.23 a	43.84 ab	26.35 ab	30.48 b	56.01 c	34.57 cd
	N_4_	24.54 a	45.65 a	26.96 a	34.29 a	58.32 b	37.67 b
	N_5_	18.45 c	41.29 cd	23.14 c	27.82 cd	55.36 c	34.89 c
	N_6_	17.34 c	39.85 de	22.41 c	26.40 d	54.66 c	34.11 cd
	*F* value	68.66**	8.23**	8.41**	33.78**	4.80**	6.49**
2018	N_0_	12.11 e	38.81 c	23.18 c	20.48 e	61.74 a	39.20 a
	N_1_	17.70 d	39.02 c	21.51 d	25.41 d	50.62 e	30.88 c
	N_2_	21.59 c	43.25 b	25.28 b	29.81 bc	54.17 cd	34.90 b
	N_3_	23.22 b	43.39 b	25.73 ab	31.74 b	54.82 c	35.17 b
	N_4_	24.84 a	45.75 a	26.67 a	35.54 a	57.95 b	38.16 a
	N_5_	18.19 d	40.30 c	22.26 cd	27.79 c	52.13 de	34.00 b
	N_6_	17.57 d	39.97 c	22.14 d	24.80 d	49.86 e	31.25 c
	*F* value	49.11**	5.92**	10.64**	44.96**	8.74**	11.89**

N_0_: no N application; N_1_: farmer urea N (180 kg ha^-1^) practice; N_2_: controlled-release N (150 kg ha^-1^) as a base; N_3_: controlled-release N (120 kg ha^-1^) as a base + urea N (30 kg ha^-1^) topdressing at panicle initiation stage; N_4_: controlled-release N (90 kg ha^-1^)+ urea N (30 kg ha^-1^) as a base + urea N (30 kg ha^-1^) topdressing at panicle initiation stage; N_5_: controlled-release N (60 kg ha^-1^)+ urea N (60 kg ha^-1^) as a base + urea N (30 kg ha^-1^) topdressing at panicle initiation stage; N_6_: controlled-release N (30 kg ha^-1^)+ urea N (90 kg ha^-1^) as a base + urea N (30 kg ha^-1^) topdressing at panicle initiation stage. NT, N transport amount; NTE, N transport efficiency; NTEC, N transport contribution rate. *, P < 0.05; **, P < 0.01. Different lowercase letters indicate significant (P < 0.05) differences among treatments under the same year.

#### 3.5.2 Nitrogen use efficiency

The effect of N fertilizer management on the NHI in 2017 was not significant, but the effect of N fertilizer management on NUE indices in the two-year experiments was significant (**Table 7**). All NUE indicators increased to different degrees in the N_2_ treatment relative to that of the N_1_ treatment, with significant increases in NBPE, NAE, NPP, and NRE of 4.60-5.18%, 17.44-17.58%, 26.84-27.13%, and 16.30-17.14%, respectively. Compared with the N_2_ treatment, all NUE indicators significantly increased and then decreased in the N_3_, N_4_, N_5_, and N_6_ treatments as the proportion of controlled-release N fertilizer in the base fertilizer decreased, with the greatest values observed in the N_4_ treatment. Increasing the proportion of available N fertilizer in the basal fertilizer beyond that of the N_4_ treatment resulted in a significant decrease in the NAE, NPP, and NRE in the N_5_ and N_6_ treatments by 8.86-13.55%, 8.88-13.58%, and 11.11-13.76%, respectively. The combination of yield characteristics (**Table 2**) showed that the rice yield and NUE could be significantly and simultaneously improved under the optimized N fertilizer reduction with controlled-release N fertilizer application (N_3_ and N_4_) treatments, especially in the N_4_ treatment, where the advantages of reducing N fertilizer application and further synergistic improvement in yield and NUE were more significant.

**Table 7:** Effects of methodical controlled-release N combined with conventional urea management on NUE of machine-transplanted hybrid *indica* rice.

Year	N treatments	NHI (%)	NBPE (kg kg^−1^)	NGPE (kg kg^−1^)	NAE (%)	NPP (kg kg^−1^)	NRE (%)
2017	N_0_	71.72 a	139.64 a	79.59 a	–	–	–
	N_1_	68.46 b	112.60 d	64.18 d	7.79 d	55.55 c	26.54 d
	N_2_	68.50 b	118.38 c	67.48 cd	15.76 c	73.08 b	36.29 b
	N_3_	68.80 b	120.37 bc	68.61 bc	21.41 b	78.72 a	42.73 a
	N_4_	69.04 b	125.38 b	71.47 b	25.37 a	82.68 a	43.68 a
	N_5_	68.55 b	120.69 bc	70.96 bc	15.89 c	73.20 b	31.15 bc
	N_6_	68.24 b	117.96 c	70.09 bc	13.40 c	70.71 b	28.89 cd
	*F* value	2.30	9.27**	8.59**	102.92**	30.64**	77.31**
2018	N_0_	72.03 a	137.18 a	78.09 a	–	–	–
	N_1_	67.28 bc	110.97 e	62.38 d	8.35 e	55.34 e	27.88 d
	N_2_	67.75 bc	116.07 d	66.02 cd	14.94 cd	71.33 cd	37.29 b
	N_3_	68.95 bc	116.15 d	67.03 bc	20.01 b	76.39 b	44.05 a
	N_4_	69.68 ab	124.81 b	70.32 b	25.79 a	82.18 a	44.18 a
	N_5_	68.15 bc	122.97 bc	68.75bc	16.93 c	73.31 bc	33.07 c
	N_6_	67.05 c	118.04 cd	68.11 bc	12.24 d	68.62 d	30.42 cd
	*F* value	4.78*	9.56**	9.32**	94.25**	24.93**	56.49**

N_0_: no N application; N_1_: farmer urea N (180 kg ha^-1^) practice; N_2_: controlled-release N (150 kg ha^-1^) as a base; N_3_: controlled-release N (120 kg ha^-1^) as a base + urea N (30 kg ha^-1^) topdressing at panicle initiation stage; N_4_: controlled-release N (90 kg ha^-1^)+ urea N (30 kg ha^-1^) as a base + urea N (30 kg ha^-1^) topdressing at panicle initiation stage; N_5_: controlled-release N (60 kg ha^-1^)+ urea N (60 kg ha^-1^) as a base + urea N (30 kg ha^-1^) topdressing at panicle initiation stage; N_6_: controlled-release N (30 kg ha^-1^)+ urea N (90 kg ha^-1^) as a base + urea N (30 kg ha^-1^) topdressing at panicle initiation stage. NHI, N harvest index; NBPE, N biomass production efficiency; NGPE, N grain production efficiency; NAE, N agronomic efficiency; NPP, N partial factor productivity; NRE, N recovery efficiency. *, P < 0.05; **, P < 0.01. Different lowercase letters indicate significant (P < 0.05) differences among treatments under the same year.

### 3.6 Correlations of yield and NUE with the photosynthetic production, root activity, and grain-filling parameters

As shown in **Table 8**, the relationship between population photosynthetic production characteristics and yield and NUE showed that except for the correlation between the LAI and NPP at the full-heading stage, the LAI and high-efficiency LAI at the full-heading stage, photosynthetic potential, and population growth rate from the jointing to full-heading stage were significantly or highly significantly positively correlated with the grain yield, filled grain number per panicle, filled grain rate, NAE, NPP, and NRE (r=0.643*-0.984**); moreover, the population growth rate from the jointing to full-heading stage was highly significantly correlated with the yield and NUE (r=0.899**–0.984**).

**Table 8:** Correlation coefficients of grain yield and NUE with population photosynthetic production, flag leaf net photosynthetic rate, root activity, and grain filling characteristics.

Index		Grain yield	Filled grains per spikelets	Filled grains rate	NAE	NPP	NRE
Photosynthetic production	LAI at full-heading stage	0.740*	0.688*	0.859**	0.669*	0.420	0.688*
	High effective LAI at full-heading stage	0.838**	0.790*	0.931**	0.781*	0.643*	0.804*
	Photosynthetic potential from jointing to full-heading stage	0.846**	0.820**	0.926**	0.789*	0.654*	0.794*
	Population growth from jointing to full-heading stage	0.984**	0.965**	0.958**	0.982**	0.899**	0.956**
*P_n_* of flag leaf after full-heading stage	0d	0.936**	0.934**	0.887**	0.957**	0.943**	0.968**
	15d	0.896**	0.900**	0.835**	0.917**	0.892**	0.881**
	30d	0.887**	0.884**	0.795*	0.894**	0.876**	0.853**
Root activity after full-heading stage	0d	0.909**	0.918**	0.869**	0.920**	0.866**	0.902**
	15d	0.935**	0.934**	0.892**	0.943**	0.906**	0.931**
	30d	0.906**	0.892**	0.827**	0.873**	0.783*	0.759*
N in stem-sheaths from full-heading to maturity stage	NT	0.857**	0.783*	0.930**	0.839**	0.716*	0.908**
	NTE	0.887**	0.815**	0.954**	0.871**	0.819**	0.937**
	NTEC	0.836**	0.739*	0.909**	0.820**	0.736*	0.915**
N in leaves from full-heading to maturity stage	NT	0.967**	0.889**	0.984**	0.931**	0.854**	0.928**
	NTE	0.913**	0.802*	0.952**	0.930**	0.714*	0.915**
	NTEC	0.893**	0.856**	0.905**	0.888**	0.702*	0.862**
Grain filling	G_mean_ of superior grains	0.658*	0.636*	0.850**	0.632*	0.626*	0.819**
	G_mean_ of inferior grains	0.965**	0.953**	0.931**	0.962**	0.875**	0.915**
	MGR of superior grains at the middle filling stage	0.664*	0.550	0.855**	0.659*	0.570	0.799**
	MGR of inferior grains at the middle filling stage	0.843**	0.852**	0.835**	0.823**	0.796**	0.824**
	MGR of inferior grains at the late filling stage	0.875**	0.839**	0.837**	0.877**	0.808**	0.827**

Coefficients of correlation between some index (except N_0_) with yield and NUE (every year number of samples is 18). LAI: leaf area index; NAE, N agronomic efficiency; NPP, N partial factor productivity; NRE, N recovery efficiency. P_n_, net photosynthetic rate; NT, N transport amount; NTE, N transport efficiency; NTEC,N transport contribution rate; G_mean_, mean filling rate during filling stage; MGR, mean grain filling rate at the certain reproductive stage. *, P < 0.05; **, P < 0.01.

Significant or highly significant positive correlations were observed between the *P_n_* of flag leaves and root activity and the yield and NUE at the full-heading stage and 15 and 30 d after full-heading (r=0.759*–0.968**). The *P_n_* values of the flag leave at the full-heading stage and root activity at 15 d after full-heading were more closely related to the synergistic improvement of yield and NUE (r=0.887**–0.968**).

Significant or highly significant positive correlations (r=0.702*–0.984**) were also observed between the NT, NTE, and NTEC of the stem sheaths (leaves) from full-heading to maturity and the yield and NUE. Among them, the NTE of stem sheaths and NT of leaves from the full-heading to maturity stage were significantly positively correlated with the yield and NUE (r=0.819**–0.984**).

Except for the insignificant correlation between the superior grains and filled grains per spikelet and NPP, the G_mean_ of the superior and inferior grains during the filling stage and the MGR of inferior grains in the middle and late stages were significantly or highly significantly positively correlated with the yield and NUE (r=0.626*–0.965**). Among these, the G_mean_ of the inferior grains throughout the filling period was more closely related to the synergistic improvement in the yield and NUE (r=0.875**–0.965**).

**Figure 4:**
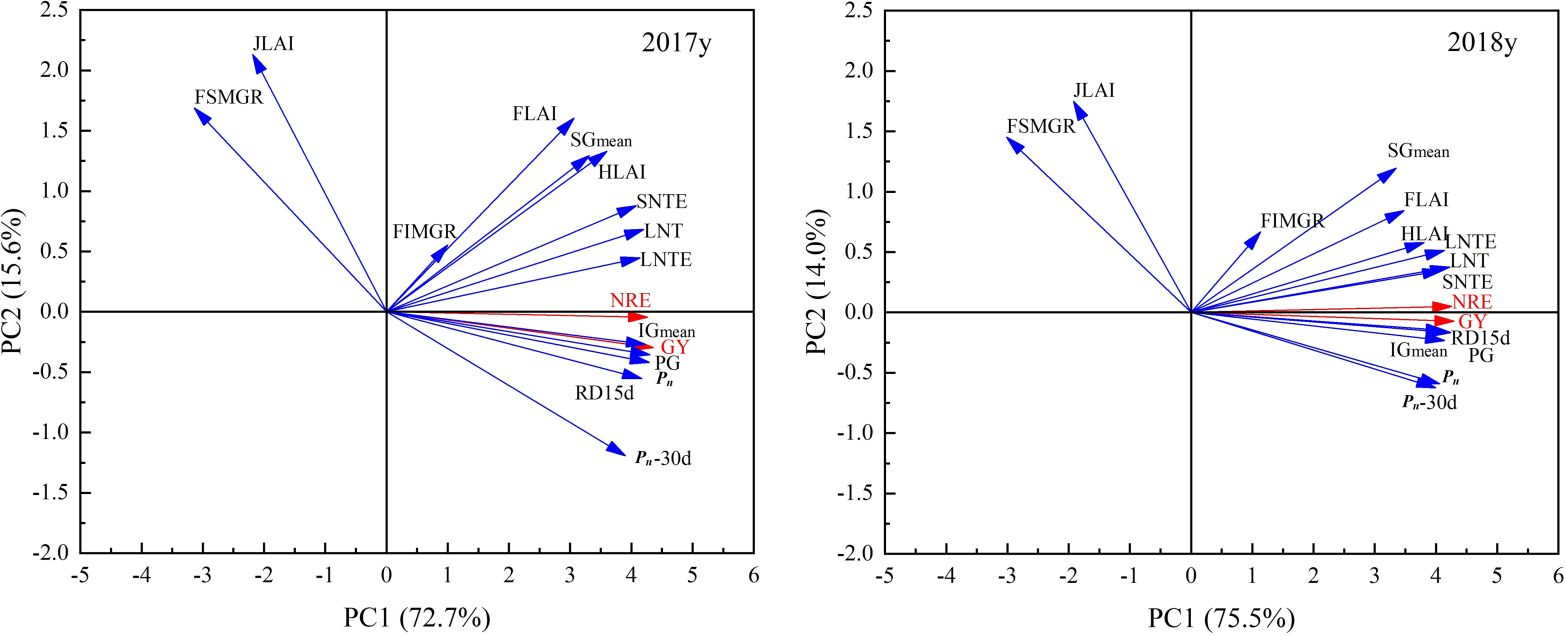
Principal component analysis of yield and NUE with photosynthetic production, root activity, and grain filling parameters. GY: Grain yield; NRE: N recovery efficiency; PG: Population growth rate from jointing to full-heading stage; JLAI: LAI at jointing stage; FLAI: LAI at full-heading stage; HLAI: high effective LAI at full-heading stage; *P_n_*: net photosynthetic rate of flag leaf at full-heading stage; *P_n_*-30d: net photosynthetic rate of flag leaf 30d after full-heading stage; RA15d: root activity 15d after full-heading stage; LNT: N transport amount in leaves from full-heading to maturity stage; LNTE: N transport efficiency in leaves from full-heading to maturity stage; SNTE: N transport efficiency in stem-sheaths from full-heading to maturity stage; SG_mean_: mean filling rate of superior grains during filling stage; IG_mean_: mean filling rate of inferior grains during filling stage; FSMGR: MGR of superior grains at the early filling stage; FIMGR: MGR of inferior grains at the early filling stage.

Further principal component analysis (**Figure 4**) showed that principal components 1 and 2 explained 88.3% and 89.5% of the total variation in paddy yield and NRE under different N fertilizer management regimes in 2017 and 2018, respectively.

**Figure 5:**
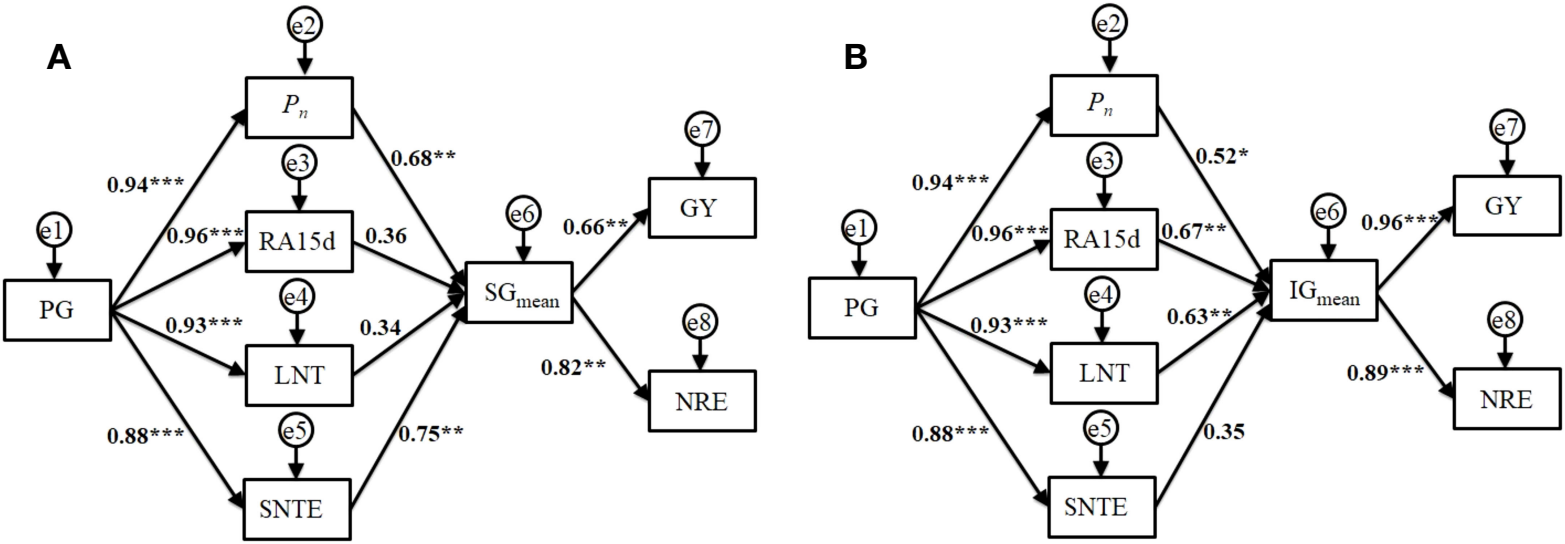
Structural equation model analysis of the main factors and regulatory pathways in superior grains **(A)** and inferior grains **(B)** affecting grain yield and NUE of machine-transplanted hybrid *indica* rice. PG: Population growth rate from jointing to full-heading stage; *P_n_*: net photosynthetic rate of flag leaf at full-heading stage; RA15d: root activity 15d after full-heading stage; LNT: N transport amount in leaves from full-heading to maturity stage; SNTE: N transport efficiency in stem-sheaths from full-heading to maturity stage; SG_mean_: mean filling rate of superior grains during filling stage; IG_mean_: mean filling rate of inferior grains during filling stage; GY, Grain yield; NRE, N recovery efficiency. The numbers represent standardized total influence coefficients *P<0.05, **P<0.01, ***P<0.001.

As shown in **Figure 5**, different N fertilizer management regimes increased the *P_n_*, root activity at 15 d after full-heading, leaf N transport amount (LNT) from full-heading to maturity, and stem sheath N transport efficiency (SNTE) from full-heading to maturity by regulating the population growth rate from jointing to the full-heading stage, which was highly significant (*P*<0.001). As shown in **Figure 5A**, the *P_n_* and SNTE had highly significant effects (*P*<0.01) on the mean filling rate of superior grains during the filling stage (SG_mean_), which in turn had a highly significant (*P*<0.01) effect on the grain yield and NUE indices (NRE). Compared with the effect on superior grains, root activity at 15 d after full-heading and LNT had a highly significant effect (*P*<0.01) on the mean filling rate of inferior grains during the filling stage (IG_mean_), and the effect of *P_n_* on IG_mean_ was significant (*P*<0.05), which in turn had a highly significant (*P*<0.001) effect on the grain yield and NRE (**Figure 5B**). This showed that each indicator had a different pathway of influence on superior and inferior grains, which in turn affected the intensity of the grain yield and NRE. Therefore, further regulation to improve IG_mean_ represents a key pathway for synergistically improving the grain yield and NRE while ensuring the filling intensity of SG_mean_.

## 4 Discussion

### 4.1 Yield and yield formation

Numerous studies have shown that although controlled-release N fertilizers present disadvantages of high cost and high price, different types of these fertilizers have a significant role in promoting the regulation of growth and development at the main growth stages of rice and ultimately achieving high yield and can also reduce N fertilizer inputs (Ke et al., 2018; Deng et al., 2021; Yu et al., 2022). Rice yield formation is closely related to population construction, photosynthetic characteristics, material accumulation and transport capacity, and yield components (Yang et al., 2014; Guo et al., 2023). Previous studies have suggested that increasing the number of panicles and grains per panicle or increasing the spikelet number of the rice population can guarantee a super-high yield (Huang et al., 2011; Zhang et al., 2013). Zhang et al. (2012) and Wei et al. (2013) have shown that a super-high yield of hybrid rice requires a sufficient number of panicles and a larger panicle type to coordinate a higher total spikelet number in the population. This study showed that the highest yield of the N_4_ treatment was obtained mainly by increasing the number of effective panicles and seed setting rate to balance the yield components and thus obtain a high yield (**Table 2**). Therefore, based on the large panicle type and sufficient storage capacity, the seed setting rate has become the main limiting factor for yield increases (Wei et al., 2013; Zhong et al., 2022); thus, the nutrient supply of controlled-release N fertilizer to the late filling of machine-transplanted rice should be guaranteed. In addition, to reduce the cost of controlled-release fertilizers and give full play to the respective advantages of controlled-release and urea-N fertilizers, researchers have studied the application techniques of slow-release N with urea-N fertilizers; however, the findings are not consistent for different regions or different application ratios (Lyu et al., 2021b; Cheng et al., 2022; Yu et al., 2022). This study also confirmed that the effects of urea-N fertilizer reduction and yield increase could be achieved by applying slow-release N fertilizer with urea topdressing. Compared with the N application rate of 180 kg/hm^2^ (N_1_ treatment), a reduction in the N application rate to 150 kg/hm^2^ and the use of controlled-release N (60%) and urea-N (20%) as a base with urea-N (20%) topdressing at the panicle initiation stage (N_4_ treatment) achieved a reduction in N fertilizer use and increase in yield (**Table 2**). However, the optimal ratio of the combined application observed here is not consistent with the results of previous studies (Lyu et al., 2021a; Zhu et al., 2021; Cheng et al., 2022). The main reason for this is that the design of the experiments considered the late topdressing of heavy-panicle varieties to study the ratios of slow-release and urea-N fertilizer, which further enriched and improved upon previous studies (Peng et al., 2014; Wu et al., 2021; AL Aasmi et al., 2022).

Previous studies have shown that the proportion of controlled-release N fertilizer is related to not only the region (Moro et al., 2015), type of controlled-release N fertilizer (Wu et al., 2021; Zhu et al., 2021), and release rate but also the rice variety chosen (Xu et al., 2021; Zhong et al., 2022). Studies on matching varieties and controlled-release N fertilizer technology have gradually become popular (Li et al., 2023). Yang et al. (2006a) pointed out that the high-yielding rice population had suitable LAI and better plant types at the heading stage, the population growth rate was high, and dry matter accumulation accounted for 70-80% of the yield after heading. However, many rice varieties have obvious regional characteristics, and the characteristics of fertilizer requirements at the main growth stages are different. In particular, the sink capacities of heavy-panicle hybrid rice and super rice are large (Zhong et al., 2022). How can the “source” and “flow” characteristics be coordinated to meet the needs of N fertilizer in the late stage under the regulation of controlled-release N fertilizer and fully exploit the potential advantages of heavy-panicle hybrid rice varieties and the advantages of controlled-release N fertilizer? The present study showed that the application technology of conventional N fertilizer for panicle fertilizer must be strengthened while optimizing the proportion of base fertilizer for controlled-release N fertilizer in machine-transplanted heavy-panicle varieties, which can improve the LAI, high-efficiency LAI, and high-efficiency leaf area rate at the full-heading stage; population photosynthetic potential and growth rate from the jointing to the full-heading stage (**Table 3**); and *P_n_* and root activity during the filling stage (**Figure 2**). Moreover, the N_4_ treatment can realize the potential advantage of heavy-panicle hybrid rice varieties. The reason may be that when using a controlled-release N fertilizer with conventional N fertilizer, the decomposition and release of N fertilizer as the fertility period progresses correspond to the critical period of fertilizer requirement before the booting stage of machine-transplanted rice; moreover, the timely supplementation of urea-N fertilizer during the booting stage when fertilizer is required is conducive to the photosynthetic production and dry matter accumulation of the plant and increases rice yield. This finding is similar to that of a previous study showing that the formation of large panicles in sufficient quantity is the basis for achieving a high rice yield (Yang et al., 2006a; Deng et al., 2021; Hou et al., 2021; Xu et al., 2021). However, not all controlled-release N fertilizer basal rates with conventional N fertilizer topdressing treatments can achieve a high yield. This study showed that when the controlled-release N fertilizer at basal rates was lower than 40% of the total N application rate, an N shortage may occur before the booting stage in machine-transplanted heavy-panicle hybrid rice (Ma and Yuan, 2015; Sun et al., 2018; Zhong et al., 2022). Although urea-N fertilizer was supplemented at the booting stage, the population photosynthetic potential and growth rate will significantly decrease from the jointing stage to the full-heading stage (**Table 3**), and the *P_n_* and root activity will decrease during the filling stage (**Figure 2**), resulting in yield reduction. In addition, this study also showed that the LAI and high-efficiency LAI at the full-heading stage and photosynthetic potential and population growth rate from the jointing to the full-heading stage were significantly positively correlated with the rice yield, filled grain number per panicle, and seed setting rate (**Table 8**). This finding further confirmed that optimizing the proportion of slow-release N base fertilizer with conventional N fertilizer topdressing was more conducive to improving the photosynthetic characteristics of machine-transplanted heavy-panicle hybrid rice and promoting the formation of a high yield.

Currently, many studies have focused on grain-filling characteristics, and the basic conclusion is that these characteristics are significantly related to the characteristics of the rice variety (Yang et al., 2006b; Wei et al., 2013; Sun et al., 2022). N fertilizer cultivation management practices can significantly regulate the process of grain-filling and yield and thus represent an important tool for further improving the yield of heavy-panicle rice varieties (Jiang et al., 2016). However, research findings on the effect of the N fertilizer application period on grain-filling are controversial (Jiang et al., 2016; Sun et al., 2022). Studies have shown that with increasing N application levels, the effective filling time increased slightly while the peak filling period did not change much, whereas, with no N application, the GR_max_ was higher than that of the normal N application (Jiang et al., 2016). Studies have also shown that postponing N topdressing is unfavorable for grain-filling (Sun et al., 2018). However, few reports have focused on the effects of controlled-release N fertilizer combined with urea-N fertilizer on grain-filling. Lyu et al. (2021b) reported that controlled-release N fertilizer combined with urea topdressing could increase the number of large and total vascular bundles at the panicle-neck internode and the number of differentiated and surviving secondary branches and spikelets, which could strengthen the source-sink relationship in rice, resulting in higher yields. This study showed that compared with the N_1_ treatment, the combined application of controlled-release N fertilizer and urea-N led to a small R_0_ and a delay in the T_max_ of grain-filling, especially when the proportion of controlled-release N fertilizer in the base fertilizer increased. Greater proportions of controlled-release fertilizer led to further delays in T_max_ (**Figure 3**; **Table 4**), which may not be conducive to grain-filling and will lead to late grain ripening (Jiang et al., 2016; Wei et al., 2018). However, the combined application of controlled-release N fertilizer and urea-N fertilizer can increase G_max_ and G_mean_, which is conducive to grain-filling, indirectly indicating a significant intermodulation effect between the treatments with controlled-release fertilizer. Optimizing the contradiction among the R_0_, T_max_, G_max_, and G_mean_ filling parameters by regulating the proportion of controlled-release N fertilizer and promoting grain plumpness of machine-transplanted heavy-panicle hybrid rice varieties may represent important methods of controlling grain-filling via the coupling of controlled-release N fertilizer and urea-N fertilizer. Yang et al. (2006b) and Sun et al. (2018) showed that increasing N fertilizer had little effect on superior grains but a greater impact on inferior grains. This study also showed that the combined application of controlled-release and urea-N fertilizer led to significant positive correlations between the yield, grain-filling rate, and MGR grain-filling characteristics of inferior grains after anthesis (**Table 8**). In particular, the average grain-filling rate of inferior grains in the middle and late stages was an important reason for the high yield and efficient utilization of controlled-release N and urea-N fertilizers. However, the mechanisms underlying the ability of the combined application of controlled-release N fertilizer and urea-N fertilizer to regulate key physiological and biochemical enzymes involved in starch synthesis and hormone levels in grains during the filling stage and the effects of such treatments on the basic basis of grain-filling remain to be further studied.

### 4.2 N use efficiency and its relationship with yield formation

Improving NUE not only improves the economic benefits but, more importantly, the N fertilizers are absorbed by the plants and remain in the soil-plant system, which reduces the pollution of the ecological environment and the harm to human health (Peng et al., 2006; Sun et al., 2023a). Wu et al. (2021) showed that under the same N application rate, N fertilizers with different controlled-release materials had different effects on NUE, mainly due to various factors, such as fertilization methods, fertilizer varieties, soil traits, and climatic conditions. Peng et al. (2014) concluded that applying controlled-release N fertilizers could effectively promote N absorption and utilization in rice, improve root activity and the panicle rate after flowering, and smoothen N transport in plants. This study showed that compared to the N_1_ treatment, reducing the N application rate to 150 kg/hm^2^ and replacing it with controlled-release N fertilizer (N_2_ treatment) significantly increased the N translocation amount, translocation rate, and translocation contribution rates of the stem sheath (leaves) from full-heading to maturity (**Table 6**). Moreover, the decrease in N fertilizer input significantly increased the NUE indexes of NBPE, NAE, NPP, and NRE but did not significantly change the NHI and NGPE (**Table 7**), which may be due to the high N accumulation in plants at maturity and insufficient N translocation from nutrient organs to grains. Deng et al. (2021) and Xu et al. (2021) showed that a “one-time application” of controlled-release N fertilizer or its combination with conventional urea could improve the NUE compared to that of conventional urea-N fertilizer management. However, this study showed that the variety of characteristics and release times of controlled-release N fertilizers should be considered. Compared with the N_2_ treatment, the N transport amount, transport rate, and transport contribution rate of stem sheath (leaves) from full-heading to maturity increased significantly and then decreased significantly in the N_3_, N_4_, N_5_, and N_6_ treatments with a reduction of the proportion of controlled-release N fertilizer in basal fertilizer, with the highest values observed in the N_4_ treatment. Therefore, when 20% panicle fertilizer is applied for heavy-panicle-type varieties, the proportion of controlled-release N fertilizer in the early stage should be more than 60% to promote better interactions with the panicle fertilizer and further improve the indicators of NUE (**Table 7**). When the basal fertilizer included all controlled-release N fertilizer or a low proportion of controlled-release N fertilizer, reductions were observed in the N transport amount, transport rate, and transport contribution rate of the stem sheath (leaves) of machine-transplanted rice from full-heading to maturity, resulting in a decrease in all indicators of NUE. This may be due to changes in population quality, such as the number of tillers, leaf growth posture, and LAI; the number of large vascular bundles and total vascular bundles in the panicle-neck internodes during rice population construction; and the different utilization efficiencies of temperature and light, which promoted changes in the N absorption and utilization rate in leaves and led to differences in N accumulation, distribution, transport, and production efficiency in various organs of plants at different growth stages.

Peng et al. (2014) and AL Aasmi et al. (2022) showed that the N absorption, transport, and NUE of hand-transplanted rice were closely related to dry matter accumulation and yield under different irrigation and controlled-release N fertilizer management patterns. Li et al. (2023) showed that N absorption, translocation, and utilization in machine-transplanted rice were closely related to the indicators of population quality for different machine-transplanted rice varieties and controlled-release N fertilizer management modes. Previous studies have shown that the yield is significantly and positively correlated with dry matter accumulation and photosynthetic potential (Huang et al., 2011; Xu et al., 2021). This study further demonstrated that the LAI and high-efficiency LAI at the full-heading stage and photosynthetic potential and population growth rate from the jointing to the full-heading stage were significantly and positively correlated with the NAE, NPP, and NRE. This study also showed that the population growth rate from the jointing to the full-heading stage was significantly and positively correlated with NUE indicators, which could be used to evaluate the NUE. However, this study only considered the jointing to full-heading stages as the entire period for calculation, and the temperature and light conditions during these stages may be inconsistent (Li et al., 2011). Subsequent research should further refine and clarify the population growth characteristics of different growth stages and clarify the key population growth stages that have significant effects on NUE (Zhang et al., 2012; Zhang et al., 2013; Deng et al., 2021). The correlation analysis in this study also showed that although the N absorption, N transport in the stem sheath, and *P_n_* and root activity during the filling stage were significantly positively correlated with NUE and yield under the combination of controlled-release N and conventional N fertilizer, these fertilizers were applied in different ways to regulate rice yield. With full controlled-release N fertilizer (N_2_ treatment), the nutrient release was slow in the early stages. This may have led to insufficient tillering or increased ineffective tillering, affecting the LAI, population photosynthetic production, and yield improvement. With the application of total conventional N fertilizer and a high proportion of conventional N fertilizer (N_1_, N_4_ and N_5_ treatments), excessive N in the early stages of vegetative growth may have led to ineffective tillering, resulting in ineffective fixation and excessive consumption of N in the leaves and stem sheaths. The lack of N nutrition in the middle stage caused uncoordinated photosynthesis, root activity, and N absorption, transport, and distribution in plants and decreased the transport rate of stem sheaths and leaves, which led to a reduction of NUE by retaining a large amount of N in the nutrient organs, thereby adversely affecting population material accumulation and yield.

The relationship between the grain-filling characteristics and NUE has rarely been reported. This study showed that the average grain-filling rate of superior and inferior grains during the entire filling stage and the average grain-filling rate of inferior grains in the middle and late stages were significantly positively correlated with the NUE indicators. In particular, the average grain-filling rate of inferior grains during the entire filling period was more closely related to the improvement in NUE. This may be because the combined application of controlled-release N and conventional N fertilizers can promote the activities of key enzymes involved in starch synthesis and N metabolism in inferior grains during the filling period to achieve synergy between grain carbon and N metabolism (Krapp et al., 2005; Sun et al., 2020b).

### 4.3 Ways of synergistically improving the yield and NUE

Several previous studies (Peng et al., 2014; Sun et al., 2020a; Deng et al., 2021; Lyu et al., 2021a; Lyu et al., 2021b; Xu et al., 2021; Yu et al., 2022) have suggested that the physiological mechanism underlying the advantage observed with applying the appropriate proportion of controlled-release N fertilizer and conventional available N fertilizer under mechanical transplanting conditions is as follows. The appropriate amount of conventional available N fertilizer in the early stage compensates for the deficiency of controlled-release N fertilizer and promotes regreening, tillering, and dry matter accumulation in rice plants, reduces ineffective tillering, and increases the tillering rate. Meanwhile, the application of controlled-release N fertilizer during the jointing, booting, and filling stages further promotes *P_n_* (Deng et al., 2021), increases the photosynthetic rate and photosynthetic potential of the population before the full-heading stage promotes dry matter accumulation from full-heading to maturity (Peng et al., 2014), and improves the canopy temperature and light characteristics (Sun et al., 2022). Moreover, the application of controlled-release N fertilizer with conventional N fertilizer can also increase the number of large and total vascular bundles as well as the number of secondary branches and spikelets that differentiate and survive and reduce the number of degraded secondary peduncles and spikelets (Lyu et al., 2021a). It can also strengthen the source-sink relationship during the filling stage, promote the coordination of carbon and N metabolism in leaves and grains, and is more conducive to N transport and redistribution to grains. To ensure a 1000-grain weight and seed setting rate, the number of filled grains per panicle was significantly increased, thus forming a high-yield population with source-sink-flow coordination and improving rice yield and NUE (Lyu et al., 2021b; Yu et al., 2022). It is important to further improve the yield and NUE of machine-transplanted hybrid rice using a controlled-release N fertilizer.

High population photosynthetic productivity and robust roots system are the basis for high yield and NUE. In this study, we showed that the application of controlled-release N fertilizer could significantly increase the *P_n_* (**Figure 1A**) and LAI (**Table 1**) and thus improve the population photosynthetic production (**Table 1**), which might be because the application of slow-release N fertilizer could increase the chlorophyll content and increase the leaf area and photosynthetic area (Wu et al., 2021; Li et al., 2023). Meanwhile, applying controlled-release N could significantly promote the root activity (**Figure 1B**) in the middle and late stages of rice and improve N translocation (**Table 6**). This might be because applying controlled-release N fertilizer could significantly increase root weight, root volume, root length and absorption area, reduce root radius, and delay root senescence (Aasmi et al., 2022). Furthermore, The present study also showed that the different slow-release N fertilizer with conventional N fertilizer treatments significantly increased the *P_n_* at the full-heading stage, root activity 15 d after the full-heading stage, N transport amount of leaves, and N translocation rate of the stem sheath from the full-heading to maturity stage by regulating the population growth rate from the jointing to the full-heading stage, which further confirmed the pathways identified in previous studies (Peng et al., 2014; Sun et al., 2020a; Deng et al., 2021; Lyu et al., 2021a; Lyu et al., 2021b; Xu et al., 2021; Yu et al., 2022). Grain-filling is the final process of rice yield formation and ultimately determines the degree of grain-filling, weight, and rice yield (Yang et al., 2006b; Jiang et al., 2016). Scarce information is available on how different controlled-release N fertilizer and conventional N fertilizer treatments affect the critical pathway of grain-filling and, thus, the yield and NUE. The present study further showed that different physiological indicators of different vegetative organs during the filling stage affected superior and inferior grains differently. The *P_n_* at the full-heading stage and N transport efficiency in the stem sheaths from the full-heading to the maturity stage had highly significant effects on the average filling rate of superior grains throughout the filling stage, significantly affecting the yield and NUE. Compared with the effect on superior grains, the root activity 15 d after the full-heading stage and the amount of N translocation in leaves from the full-heading to maturity stage had a highly significant effect on the average filling rate of inferior grains throughout the filling period and then significantly affected the yield and NRE. To guarantee the filling strength of the mean filling rate of the superior grain during the filling stage, further regulation is required to increase the mean filling rate of the inferior grain during the filling stage and synergistically improve the yield and NRE, which further enriches and completes the regulation pathway detailed in previous studies. In addition, this study clearly shows that treatment with controlled-release N (60%) and urea-N (20%) as a base with urea-N (20%) as topdressing at the panicle initiation stage can promote the synergistic improvement of the yield and NUE of machine-transplanted heavy-panicle hybrid rice. Methods of realizing fertilizer requirement characteristics of different varieties and optimizing the slow-release N fertilizer application technology must be further investigated. Moreover, whether slow-release N fertilizer application technology can synergistically improve yield and NUE while reducing greenhouse gas emissions in rice fields requires further study.

## 5 Conclusion

This study showed that the optimized controlled-release N base fertilizer ratios and urea topdressing have significant effects on the photosynthetic production characteristics, root activity, N translocation, and grain-filling parameters of machine-transplanted hybrid *indica* rice, with particularly strong effects on the G_mean_ of inferior grains, grain yield, and NUE indices (NAE, NPP, and NRE). That the treatment of controlled-release N (60%) and urea-N (20%) as a base and urea-N (20%) as topdressing at the panicle initiation stage can optimize the source-sink relationship in machine-transplanted hybrid *indica* rice, resulting in a synergistically higher grain yield and NUE.

## Data availability statement

The original contributions presented in the study are included in the article/**Supplementary Material**, further inquiries can be directed to the corresponding author/s.

## Author contributions

YYS: Data curation; investigation; writing-original draft. XY: Formal analysis; investigation. KC: Formal analysis; software. HW: Formal analysis; validation. YL: Methodology. CG: Validation; visualization. ZW: Visualization. CS: Methodology; investigation. YY: Formal analysis; software. YW: Validation; software. XZ: investigation; ZY: writing-review and editing. JM: project administration; writing-review and editing. YJS: Funding acquisition; project administration; writing-original draft. All authors contributed to the article and approved the submitted version.
