# Supplementary material for: Improving the yield and nitrogen use efficiency of hybrid rice through rational use of controlled-release nitrogen fertilizer and urea topdressing
Source: Front Plant Sci. 2023 Aug 24;14:1240238. doi: 10.3389/fpls.2023.1240238 (PMC10484103; doi:10.3389/fpls.2023.1240238)
Supplement: Supplementary file 1 [file DataSheet_1.docx]

***Supplementary Material***

**Supplementary TABLE 1** Relative percentage increase in yield and yield formation of machine-transplanted hybrid *indica* rice under controlled-release N combined with urea-N management relative to N_0_ treatment (%)

| Year | N treatments | Grain yield | Effective panicles | Filled spikelets | High effective LAI at full-heading  stage | Photosynthetic potential from jointing to full-heading stage | Population growth rate from jointing to full-heading stage |
| --- | --- | --- | --- | --- | --- | --- | --- |
| 2017 | N_0_ | 0.00 | 0.00 | 0.00 | 0.00 | 0.00 | 0.00 |
|  | N_1_ | 16.31 | 15.97 | 4.23 | 70.59 | 66.55 | 1.44 |
|  | N_2_ | 27.50 | 19.44 | 6.56 | 65.69 | 58.93 | 14.09 |
|  | N_3_ | 37.36 | 21.47 | 13.41 | 79.90 | 68.76 | 28.65 |
|  | N_4_ | 44.26 | 25.48 | 14.58 | 127.45 | 88.68 | 33.85 |
|  | N_5_ | 27.72 | 17.52 | 9.48 | 52.45 | 59.65 | 18.42 |
|  | N_6_ | 23.38 | 15.92 | 7.58 | 49.02 | 55.19 | 6.28 |
|  |  |  |  |  |  |  |  |
| 2018 | N_0_ | 0.00 | 0.00 | 0.00 | 0.00 | 0.00 | 0.00 |
|  | N_1_ | 17.77 | 11.96 | 8.80 | 67.96 | 68.05 | 2.34 |
|  | N_2_ | 26.50 | 16.04 | 11.24 | 64.08 | 66.46 | 14.72 |
|  | N_3_ | 35.48 | 16.62 | 15.98 | 101.94 | 85.84 | 28.32 |
|  | N_4_ | 48.11 | 23.82 | 19.45 | 124.76 | 91.06 | 35.15 |
|  | N_5_ | 30.03 | 15.56 | 13.09 | 52.91 | 65.30 | 14.98 |
|  | N_6_ | 21.70 | 13.71 | 11.32 | 46.60 | 60.03 | 5.20 |

N_0_: no N application; N_1_: farmer urea N (180 kg ha^-1^) practice; N_2_: controlled-release N (150 kg ha^-1^) as a base; N_3_: controlled-release N (120 kg ha^-1^) as a base + urea N (30 kg ha^-1^) topdressing at panicle initiation stage; N_4_: controlled-release N (90 kg ha^-1^)+ urea N (30 kg ha^-1^) as a base + urea N (30 kg ha^-1^) topdressing at panicle initiation stage; N_5_: controlled-release N (60 kg ha^-1^)+ urea N (60 kg ha^-1^) as a base + urea N (30 kg ha^-1^) topdressing at panicle initiation stage; N_6_: controlled-release N (30 kg ha^-1^)+ urea N (90 kg ha^-1^) as a base + urea N (30 kg ha^-1^) topdressing at panicle initiation stage.*, *P* < 0.05; **, *P* < 0.01. Different lowercase letters indicate significant (*P* < 0.05) differences among treatments under the same year.

**Supplementary TABLE 2** Relative percentage increase in net photosynthetic rate and root activity during filling stage of machine-transplanted hybrid *indica* rice under controlled-release N combined with urea-N management relative to N_0_ treatment (%)

| Year | N treatments | Net photosynthetic  Days after full-heading(d) | | |  | Root activity  Days after full-heading(d) | | |
| --- | --- | --- | --- | --- | --- | --- | --- | --- |
|  |  | 0 | 15 | 30 |  | 0 | 15 | 30 |
| 2017 | N_0_ | 0.00 | 0.00 | 0.00 |  | 0.00 | 0.00 | 0.00 |
|  | N_1_ | 27.70 | 29.51 | 17.21 |  | 16.62 | 25.45 | 14.88 |
|  | N_2_ | 44.38 | 54.50 | 50.03 |  | 42.54 | 57.47 | 53.94 |
|  | N_3_ | 61.74 | 83.94 | 86.27 |  | 88.00 | 88.32 | 101.57 |
|  | N_4_ | 63.09 | 84.59 | 78.07 |  | 83.45 | 91.60 | 107.14 |
|  | N_5_ | 40.81 | 64.17 | 58.76 |  | 55.71 | 68.22 | 69.48 |
|  | N_6_ | 37.25 | 42.00 | 47.93 |  | 33.45 | 36.79 | 21.35 |
|  |  |  |  |  |  |  |  |  |
| 2018 | N_0_ | 0.00 | 0.00 | 0.00 |  | 0.00 | 0.00 | 0.00 |
|  | N_1_ | 14.07 | 28.81 | 23.62 |  | 7.84 | 28.61 | 32.38 |
|  | N_2_ | 39.19 | 53.20 | 49.06 |  | 35.89 | 49.38 | 49.00 |
|  | N_3_ | 54.59 | 73.73 | 69.02 |  | 68.58 | 72.13 | 110.93 |
|  | N_4_ | 53.76 | 69.92 | 70.85 |  | 67.33 | 74.36 | 113.67 |
|  | N_5_ | 35.61 | 61.09 | 55.00 |  | 43.22 | 50.10 | 97.41 |
|  | N_6_ | 33.67 | 39.88 | 39.54 |  | 15.00 | 33.56 | 53.98 |

N_0_: no N application; N_1_: farmer urea N (180 kg ha^-1^) practice; N_2_: controlled-release N (150 kg ha^-1^) as a base; N_3_: controlled-release N (120 kg ha^-1^) as a base + urea N (30 kg ha^-1^) topdressing at panicle initiation stage; N_4_: controlled-release N (90 kg ha^-1^)+ urea N (30 kg ha^-1^) as a base + urea N (30 kg ha^-1^) topdressing at panicle initiation stage; N_5_: controlled-release N (60 kg ha^-1^)+ urea N (60 kg ha^-1^) as a base + urea N (30 kg ha^-1^) topdressing at panicle initiation stage; N_6_: controlled-release N (30 kg ha^-1^)+ urea N (90 kg ha^-1^) as a base + urea N (30 kg ha^-1^) topdressing at panicle initiation stage.*, *P* < 0.05; **, *P* < 0.01. Different lowercase letters indicate significant (*P* < 0.05) differences among treatments under the same year.

**Supplementary TABLE 3** Relative percentage decrease in N transport amount in stem-sheaths and leaves from full-heading to maturity stage and NUE of machine-transplanted hybrid *indica* rice under controlled-release N combined with urea-N management relative to N_4_ treatment (%)

| Year | N treatments | N transport amount | |  | NUE | | |
| --- | --- | --- | --- | --- | --- | --- | --- |
|  |  | stem-sheaths | leaves |  | NAE | NPP | NRE |
| 2017 | N_0_ | -52.08 | -40.89 |  | - | - | - |
|  | N_1_ | -24.78 | -21.06 |  | -17.58 | -27.13 | -17.14 |
|  | N_2_ | -13.12 | -16.91 |  | -9.61 | -9.60 | -7.39 |
|  | N_3_ | -5.34 | -11.11 |  | -3.96 | -3.98 | -0.95 |
|  | N_4_ | 0.00 | 0.00 |  | 0.00 | 0.00 | 0.00 |
|  | N_5_ | -24.82 | -18.87 |  | -9.48 | -9.48 | -12.53 |
|  | N_6_ | -29.34 | -23.01 |  | -11.97 | -11.97 | -14.79 |
|  |  |  |  |  |  |  |  |
| 2018 | N_0_ | -51.25 | -42.37 |  | - | - | - |
|  | N_1_ | -28.74 | -28.50 |  | -17.44 | -26.84 | -16.30 |
|  | N_2_ | -13.08 | -16.12 |  | -10.85 | -10.85 | -6.89 |
|  | N_3_ | -6.52 | -10.69 |  | -5.78 | -5.79 | -0.13 |
|  | N_4_ | 0.00 | 0.00 |  | 0.00 | 0.00 | 0.00 |
|  | N_5_ | -26.77 | -21.81 |  | -8.86 | -8.87 | -11.11 |
|  | N_6_ | -29.27 | -30.22 |  | -13.55 | -13.56 | -13.76 |

N_0_: no N application; N_1_: farmer urea N (180 kg ha^-1^) practice; N_2_: controlled-release N (150 kg ha^-1^) as a base; N_3_: controlled-release N (120 kg ha^-1^) as a base + urea N (30 kg ha^-1^) topdressing at panicle initiation stage; N_4_: controlled-release N (90 kg ha^-1^)+ urea N (30 kg ha^-1^) as a base + urea N (30 kg ha^-1^) topdressing at panicle initiation stage; N_5_: controlled-release N (60 kg ha^-1^)+ urea N (60 kg ha^-1^) as a base + urea N (30 kg ha^-1^) topdressing at panicle initiation stage; N_6_: controlled-release N (30 kg ha^-1^)+ urea N (90 kg ha^-1^) as a base + urea N (30 kg ha^-1^) topdressing at panicle initiation stage. NAE: N agronomic efficiency; NPP: N partial factor productivity; NRE: N recovery efficiency. *, *P* < 0.05; **, *P* < 0.01. Different lowercase letters indicate significant (*P* < 0.05) differences among treatments under the same year.
